# Magnetic Resonance Imaging (MRI) to Study Striatal Iron Accumulation in a Rat Model of Parkinson’s Disease

**DOI:** 10.1371/journal.pone.0112941

**Published:** 2014-11-14

**Authors:** Ana Virel, Erik Faergemann, Greger Orädd, Ingrid Strömberg

**Affiliations:** 1 Department of Integrative Medical Biology, Umeå University, Umeå, Sweden; 2 Department of Radiation Sciences, Umeå University, Umeå, Sweden; National Health Research Institutes, Taiwan

## Abstract

Abnormal accumulation of iron is observed in neurodegenerative disorders. In Parkinson’s disease, an excess of iron has been demonstrated in different structures of the basal ganglia and is suggested to be involved in the pathogenesis of the disease. Using the 6-hydroxydopamine (6-OHDA) rat model of Parkinson’s disease, the edematous effect of 6-OHDA and its relation with striatal iron accumulation was examined utilizing *in vivo* magnetic resonance imaging (MRI). The results revealed that in comparison with control animals, injection of 6-OHDA into the rat striatum provoked an edematous process, visible in T2-weighted images that was accompanied by an accumulation of iron clearly detectable in T2*-weighted images. Furthermore, Prussian blue staining to detect iron in sectioned brains confirmed the existence of accumulated iron in the areas of T2* hypointensities. The presence of ED1-positive microglia in the lesioned striatum overlapped with this accumulation of iron, indicating areas of toxicity and loss of dopamine nerve fibers. Correlation analyses demonstrated a direct relation between the hyperintensities caused by the edema and the hypointensities caused by the accumulation of iron.

## Introduction

Brain iron accumulation is a normal consequence of ageing. In neurodegenerative disorders this accumulation of iron seems to be augmented and has been proposed as a possible cause of neural death [1]–[3]. The mechanisms behind anomalous brain iron metabolism are not well understood. Previously, it was thought that abnormal accumulation of iron was a secondary event of neurodegeneration, but today several studies corroborate that abnormal brain iron deposition can originate from different sources such as misregulation of iron transport and storage or transcriptional modifications [1], [3]–[5]. Ferrous iron can react with H_2_O_2_ via the Fenton reaction and lead to the formation of ferric iron (Fe^+3^) and a hydroxyl radical. The hazard of the Fenton reaction is that the resulting reactive oxygen species (ROS) could participate in a cascade of events that lead to tissue oxidative stress [6]–[8]. Non-heme brain iron is mostly found in the ferric (+3) state bound to ferritin, and only a small amount of free intracellular ferrous iron is available [9], [10]. Under non-pathological conditions cells have different mechanisms to protect themselves from the formation of these hazardous radicals. However, with age or in situations of abnormal iron accumulation, the availability of ferrous iron and formation of ROS is normally augmented [4], [9].

In Parkinson’s disease, an abnormal iron overload is present in the basal ganglia [11], [12]. Indeed, new advances in the development of neuroprotective drugs to palliate this disorder are focused on the use of different iron chelators to remove toxic iron from brain tissue [13]. However, while much attention has been paid to the toxic effect of iron in the substantia nigra, little is known about the origin of iron deposition in other structures of the dopaminergic system. Iron can be taken up by neurons via the transferrin receptor [14] and then be axonally transported to iron-rich areas in the brain [15]. In dopaminergic neurons an excess of iron might constitute an extra threat since iron has the ability to react with dopamine and produce free radicals [4], [16]. In parkinsonian patients, an inverse relationship between striatal iron levels and dopamine concentration has been found, suggesting a retrograde transport of iron from the nerve terminals in the striatum to the cell soma in the substantia nigra [17]. In fact, it has been postulated that degeneration in Parkinson’s disease might first occur at the level of the striatal dopamine nerve fibers, and consequently trigger a slow retrograde degeneration of the dopamine neurons situated in the substantia nigra [18]. Therefore, it is important to investigate the possible causes of early dopamine nerve terminal loss in the striatum to clarify possible origins of this disorder.

In animal models of Parkinson's disease, iron levels are increased in the substantia nigra of N-methyl-4-phenyl-1,2,3,6-tetrahydropyridine (MPTP) and 6-hydroxydopamine (6-OHDA) injected animals [19]–[22]. Furthermore, high concentrations of iron can promote alpha-synuclein aggregation in dopaminergic cells, leading to the formation of Lewy bodies [23]. Novel studies have demonstrated that knockout tau mice accumulate iron in the substantia nigra and are more prone to develop parkinsonism, suggesting a role of tau protein as an iron export mediator [24]. However, due to the difficulties in diagnosing Parkinson’s disease at early stages, most studies regarding iron accumulation and dopamine degeneration have been performed postmortem. In animal models, studies have been focused on the accumulation of iron in the substantia nigra after complete depletion of the dopamine neurons, thus, only mimicking late stages of the disorder [19], [25]. It is therefore of great importance to investigate if iron overload can also be found at earlier phases of neurodegenerative diseases in other structures of the basal ganglia than in the substantia nigra to be able to hamper its toxic effects.

Magnetic resonance imaging (MRI) has emerged as one of the most promising techniques to study brain iron accumulation in neurodegenerative disorders [26]–[28]. Image contrast is characterized by proton density, together with the longitudinal and transverse proton relaxation. The effect of iron on the longitudinal relaxation is weak and will not be considered here. There exists three transverse relaxation rates, characterized by the transverse relaxation times T2, T2* and T2’, and related by 1/T2* = 1/T2+1/T2’. T2 and T2* governs the relaxation in spin-echo and gradient echo sequences, respectively and differ in that the effect of local magnetic field inhomogeneities are removed in the spin-echo sequence. Labile iron is hardly detectable in MRI. On the contrary, ferritin aggregates or hemosiderin greatly shortens the relaxation time of water protons resulting in hypointensities in T2- and T2*-weighted images (WI) [10], [28]–[30]. Thus, each of these relaxation times can, and has indeed been used to characterize iron accumulation in biological tissue disorders [28], but no consensus on the best method has yet been reached, because of the disadvantages associated with each approach. The iron dependence on T2 has been used to some extent but, since also other factors, such as water content and biological structure, also affect T2, this relationship is not always easily exploited. The effect of iron on T2* is stronger due to the sensitivity to both the reversible and the irreversible effects of iron on the proton relaxation and is therefore considered a more sensitive and robust method to identify iron stores [29], [31]. In fact, T2* analysis have been suggested as a biomarker to calculate iron deposition in Parkinsońs disease [32]. On the downside, T2* is also affected by other sources of magnetic field variation, e.g. air-filled cavities that makes gradient echo imaging difficult in some parts of the brain, such as the substantia nigra. The susceptibility effect of the iron is also more spatially spread so that the affected area will be less defined in T2*-WI.

In parkinsonian patients, MRI analyses at advanced stages of the disease have shown evidence of iron accumulation based on shortening of relaxation times [32]–[34]. In animal models, there are only a few MRI reports on the neurotoxic effect of 6-OHDA or MPTP. These reports are basically focused on the use of T2-WI, to study the edematous effect of 6-OHDA [35], [36]. Attempts have also been made to use T2 contrast to detect iron accumulation in the nigrostriatal structures, however, the results are not consistent, probably due to the small effect of iron on T2 [37], [38]. While most studies to date have focused on iron accumulation in the substantia nigra, the present work is using MRI to monitor the effects of the neurotoxin 6-OHDA on striatal iron accumulation using T2*- and T2-WI. Unilateral injection of 6-OHDA into the striatum of young rats is commonly used as a model for early Parkinson’s disease. When 6-OHDA is injected into the striatum, it is specifically taken up by catecholaminergic neurons and then autoxidized, producing reactive oxygen species (ROS) [39]. As a consequence, a slow retrograde degeneration of the dopamine system occurs which lasts over several weeks [40].

Here, the hemiparkinsonian rat model was used to monitor by MRI the effect of 6-OHDA on edema formation and its relation with brain iron accumulation in the striatum. Dopamine degeneration as well as microglia infiltration were also evaluated using immunohistochemistry, and the presence of iron was evaluated using Perls Prussian blue.

## Materials and Methods

### Animals and surgery

Female Sprague-Dawley rats were used in this study (*n* = 19). All animal experiments were performed in accordance with international accepted guidelines, and approved by the local ethics committee, Umeå Ethics Committee for Animal Studies (approval number A68/12). Animals were housed under a 12: 12 h light/dark cycle and provided with food and water *ad libitum.* All efforts were made to minimize animal suffering.

### Dopamine lesions

Rats were anesthetized with isoflurane in O_2_ (Baxter Medical AB, Kista, Sweden) and subjected for stereotaxic injections of 6-OHDA (*n* = 10) or vehicle (*n* = 9) into the right dorsal striatum. The following coordinates were used: 1.0 mm anterior, 2.8 mm lateral to bregma, and 5.0 mm below the dura. 6-OHDA injected animals received a dose of 20 µg of 6-OHDA, dissolved in 4 µL saline (0.9% NaCl) containing 0.02% ascorbic acid (pH 3.7). Control animals were injected with vehicle, consisting of 4 µL saline containing 0.02% ascorbic acid. Injections were performed at a rate of 1 µL/min using a Hamilton syringe with a 26 gauge stainless steel needle. The cannula was left in place for 4 min following injection.

### *In vivo* MRI

MRI proton imaging experiments were performed at 9.4 T using a Bruker BioSpec 94/20 scanner equipped with a BG12S gradient set and running Paravision 5.1 software (Bruker, Ettlingen, Germany). Animals were anesthetized with isoflurane in O_2_ and scanned utilizing a 40 mm quadrupolar volume coil (Bruker). Temperature and breathing rate were monitored during the course of the experiments using a SA physiological monitoring system (SA Instruments, Inc; Stony Brook, USA). Scans were performed 2 days, 1 week, 3 weeks, and 4 weeks after the intrastiatal injection. To calculate hypointensities and hyperintensities in the striatum T2 and T2* WI were executed: T2-WI were performed using a spin-echo sequence (RARE, TR/TE = 2500/33 ms, Matrix = 256, number of excitations = 4). For T2*-WI a gradient echo sequence was used (MGE, TR/TE = 1500/4, 10, 16, 22, 28, 34, 40, 46, 52, 58, 64, 70 ms, Matrix = 96, number of excitations = 2) from which T2*-maps were subsequently calculated. For both sequences a slice thickness of 0.30 mm with no interslice gap and a field of view of 2.88 cm, was used. The low resolution used for the creation of T2* parametric maps was motivated by the need of better signal to noise ratio for the fitting procedure. The rather low resolution will give rise to larger partial volume effects. However, we do not believe that this will affect the comparative analysis, especially when considering that the susceptibility contribution to T2* is less localized, therefore the effect will be more large-spread for T2* than for T2. For picture illustration an additional gradient echo sequence was utilized with higher in-plane resolution and thicker slices (TR/TE = 1500/22 ms, Matrix 256, Number of excitations = 2, slice thickness 0.53 mm, no interslice gap, Field of view = 3.00 cm). To analyze hypointensities in the substantia nigra a gradient echo sequence was used (TR/TE = 1500/10 ms, Matrix 256, Number of excitations = 2, slice thickness 0.53 mm, no interslice gap, Field of view = 3.00 cm).

### Image analysis and quantifications in the striatum

**Figure 1:**
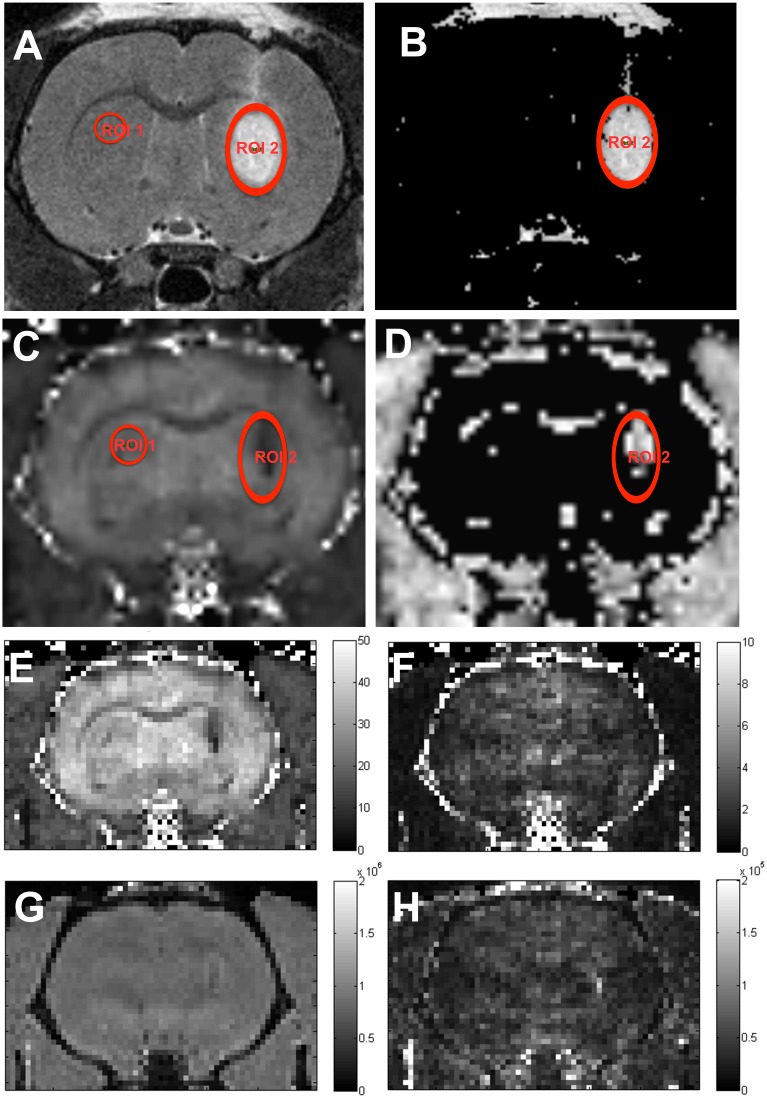
Image processing and ROI selection. T2-WI (A) and T2*-maps (C) were processed into thresholded intensity (B) and T2* maps (D) to calculate areas of affected relaxation times. A control (ROI1) was selected in the unprocessed image and the mean intensity value was used as a reference to select affected pixels (B, D). Only voxels within ROI2 were included in the analysis. Also included are the non-smoothed results of the fit, presented as T2* parametric maps (E) and its standard deviation (F) (scale bars in ms) and intensity (G) and its standard deviation (H) (scale bars in arbitrary units). Note that although figs. A–D are smoothed (Paravision default for image presentation), the ROI statistics are calculated on the non-smoothed data. High resolution T2*-WI of the same animal are represented in Fig. 7.

Areas of affected relaxation times were quantified from T2-WI and T2*-WI using Paravision software 5.1 (Bruker) and expressed as pixel area (pu^2^). The affected volumes were calculated similar to the method described in [35] by selecting voxels with intensities lying above (hyperintensities) or below (hypointensities) a threshold determined from the mean value for a control area (Fig. 1, ROI1). The cutoffs that gave the best signal to noise ratio were selected to process the pictures. Only voxels within a defined region of interest (Fig. 1, ROI2) were considered in the analysis. Total hypointense and hyperintense areas were calculated for each slice (0.3 mm thickness). When depicting the intensity values at each distance, each value represented the sum of two contiguous slices (0.6 mm).

#### T2-WI in the striatum (Fig. 1A–B).

The control area (ROI1, 0.02 cm^2^) was located in the contralateral striatum. Hyperintense voxels were defined as voxels within ROI2 having an intensity larger than 150% of the mean value in the control. For hypointense voxels the corresponding threshold was set to 65% of the control mean.

#### T2* maps in the striatum (Fig. 1C–D).

T2* maps were calculated by fitting the voxel intensities to a single exponential decay for the different echo times, I = I0*exp(-TE/T2*), using the fitting routines in Paravision 5.1 (Fig. 1E–H). The control area was located in the contralateral striatum (ROI1, 0.02 cm^2^) and voxels with T2*-values lower than 75% of the mean of the control were defined as affected. This method is expected to give more accurate estimates compared to using voxel intensities for a single echo time. According to the equation above, voxels with low values of T2* will give rise to hypointensities in T2*-WI and, in this respect, we will use the term hypointensities both to denote voxels of lower intensity in T2*-WI and voxels of low T2* values in T2*-maps.

#### T2* WI in substantia nigra

The control ROI was drawn outside the substantia nigra. The region corresponding to the substantia nigra was located by correlating the MRI pictures with the atlas of *Paxinos et al,* and voxels in these regions were considered affected if the T2*-values were lower than 75% of the mean of the control.

### Statistical analysis

To study hyperintensities caused by the presence of edema, non-parametric tests were used. Differences in hyperintensities between animal groups at each time point or position were analyzed using the Mann-Whitney test. To detect differences between distances from the injection site within each group, Kruskal-Wallis test was performed followed by a set of different Mann-Whitney tests using Bonferroni correction. To study differences in hyperintense area within each group at different time points Friedman Test was performed followed by Wilcoxon signed-rank tests.

To study differences in hypointensities between different distances from the injection, two-factor ANOVA was first performed to assess interactions between animal treatments (control/6-OHDA) and distances. Student’s *t*-test was performed to determine differences in hypointensities at each distance between animal treatments. One-way ANOVA followed by Bonferroni *post hoc* were performed to detect differences in hypointensities at each distance within each group. To determine differences in hypointensities between 6-OHDA and control injected animals at each time point, mixed-design ANOVA was used. Student’s *t*-test was performed to determine differences in hypointensities at each time point between animal groups. One-way repeated measurements ANOVA followed by pairwise comparisons were performed to detect differences in hypointensities within each group. All results are expressed as mean values ± SEM. The significant level was set at p<0.05.

Pearson correlation and linear regression analysis were performed to correlate hyperintense and hypointense values. Scatter plots including the best linear fit were performed using Kaleidagraph 4.0 (Synergy software).

### Histological analysis

Two days (n = 8) or 4 weeks (n = 11) after the striatal dopamine lesion, the rats were anesthetized with pentobarbital and transcardially perfused with Ca^+2^-free Tyrode solution, followed by 4% paraformaldehyde in 0.1 M phosphate buffer (pH 7.4). The brains were then removed and postfixed in 4% paraformaldehyde in phosphate buffer (pH 7.4) overnight. Afterwards, the brains were stored at 4°C in 10% sucrose solution 0.01% NaN_3_ in phosphate buffer (pH 7.4) until the brains were processed for sectioning (between 4 days and 3 months). Sucrose solution was changed regularly. The brains from all animals in the study (6-OHDA and controls) were frozen using CO_2_ and axial sections (14 µm) from the striatum were collected. Sections were thawed onto gelatin-coated glass slides, and rinsed for 15 min in 0.1 M phosphate buffered saline (PBS; pH 7.4), prior to Perls staining or antibody incubations.

#### Perls Prussian blue staining and ED1 immunohistochemistry

To detect ferric iron in the sectioned brains, the Prussian blue method with DAB enhancement was used. Briefly, tissue sections from all animals were incubated in PBS (pH 7.4) for 3×5 min. Afterwards, endogenous peroxidase was blocked by incubating the samples in 1% H_2_O_2_ in PBS (pH 7.4) for 15 min and then washed 3×5 min in PBS (pH 7.4). The sections were then incubated in a fresh solution of equal parts of 2% HCl mixed with 2% potassium ferrocyanide in PBS (pH 7.4) for 45 min and then rinsed for 3×5 min in PBS (pH 7.4). DAB enhancement was performed by incubating in diaminobenzidine (DAB) solution (Sigma, Stockholm, Sweden) for 20 min. Samples were then washed 3×5 min in PBS (pH 7.4). After Prussian blue staining, the same sections were processed for immunohistochemical detection of active microglia. Microglial cells were visualized using antibodies raised against ED1 (mouse anti-rat, dilution 1∶100 Millipore, Solna, Sweden) and secondary antibodies Alexa Fluor A488-conjugated antibodies (goat anti-mouse, diluted 1∶200, Molecular Probes, Oregon, USA). Sections were cover-slipped in 90% glycerol in PBS (pH 7.4).

#### Immunoglobulin G (IgG) immunohistochemistry

To study the integrity of the blood-brain barrier (BBB) IgG immunohistochemistry was performed 2 days after the dopamine lesion [41]. Tissue sections were incubated for 1 hour at room temperature with antibodies specific for rat IgG (Alexa Fluor A594 rabbit anti-rat IgG, Invitrogen) dilution 1∶200 in 0.3% Triton-X-100 in PBS (pH 7.4). Sections were rinsed 3×15 min in PBS (pH 7.4) and cover-slipped in 90% glycerol in PBS (pH 7.4).

#### Tyrosine hydroxylase (TH) immunohistochemistry

Dopamine denervation was evaluated using primary antibodies raised against tyrosine hydroxylase (TH; rabbit anti-rat, dilution 1∶300, Millipore, Solna, Sweden), and secondary antibodies Alexa Fluor A594 (goat anti-rabbit, dilution 1∶500 Molecular Probes, Oregon, USA). All antibodies were diluted in 0.3% Triton-X-100 in PBS (pH 7.4). Primary antibody incubations were performed for 48 hours at 4°C and secondary antibody incubations for 1 hour at room temperature. Preceding TH secondary antibody incubation, unspecific bindings were blocked with 5% goat serum for 15 min. Between antibody incubations, sections were rinsed 3×15 min in PBS (pH 7.4). Sections were cover-slipped in 90% glycerol in PBS (pH 7.4).

#### Giemsa staining

To evaluate the presence of hemorrhages at 2 days after the dopamine lesion, the sections were incubated in Giemsa solution for 5 min, and then washed in PBS. The sections were then mounted in 90% glycerol in PBS (pH 7.4).

Images were captured with Jenoptic, (Jena, Germany) or Retiga 4000RV CCD camera (Q-Imaging, Surrey, BC, Canada).

## Results

### Hyperintensities in the striatum

**Figure 2:**
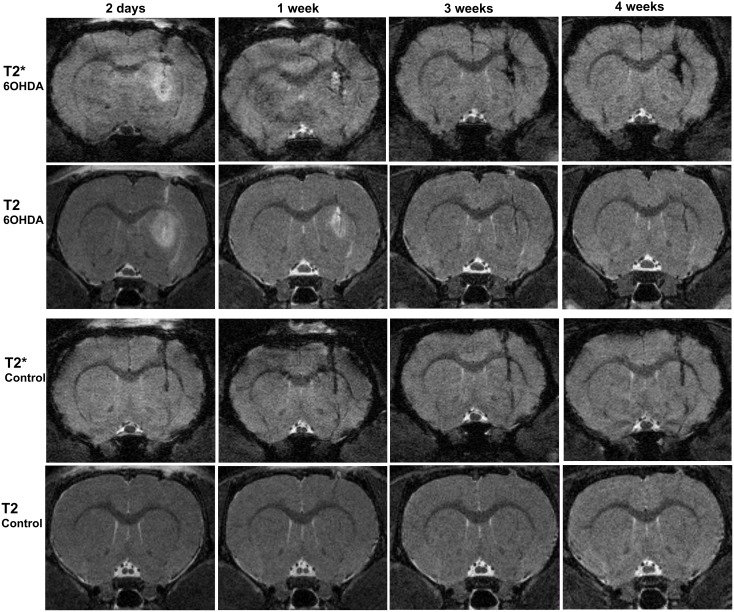
High resolution T2*- and T2 WI at different time points after striatal injections. Upper panels represent 6-OHDA injected animals and lower panels control animals. At two days a strong hyperintensity caused by the presence of edema is visible in T2 and T2*-WI in 6-OHDA- but not in control animals. T2*-WI from 1–4 weeks demonstrated a stronger and more widespread hypointense signal in 6-OHDA injected animals comparing with control animals.

**Figure 3:**
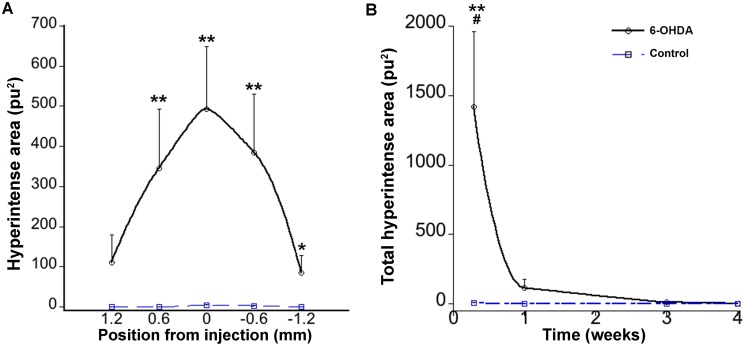
Hyperintense evaluations from T2-WI after striatal injections. A) Hyperintense distributions at 2 days postlesion in the striatum expressed as distances anterior (positive) and posterior (negative) to the injection site. Each point represents the summation of hyperintensities from two 0.3 mm-thick contiguous images. There are significant differences between control and 6-OHDA treated animals. B) Time course evolution of total hyperintense areas also differs from control animals. Continuous line represents 6-OHDA injected animals and broken line represents control animals. (*p<0.05, **p<0.01 compared between 6-OHDA and control animals, ^#^p<0.05 compared between time points within 6-OHDA group).

Two days after the striatal 6-OHDA injection T2-WI revealed a strong hyperintense signal around the site of injection, whereas this phenomenon was almost not noticeable in control animals (Figs. 2 and 3A). Mann-Whitney test revealed a significant difference in hyperintensities at 2 days between 6-OHDA and control animals (U = 0.000, *z* = −2.739 p<0.01). The hyperintensity appeared to be maximum at the injection site and spread at least 1 mm anterior-posteriorly (Fig. 3A). When comparing hyperintense values at different time points in the 6-OHDA injected animals, the peak value was observed at day two followed by a progressive decrease. At one week the hyperintense area had faded and it was vanished at three weeks (Fig. 3B).

### Hypointensities in the striatum

**Figure 4:**
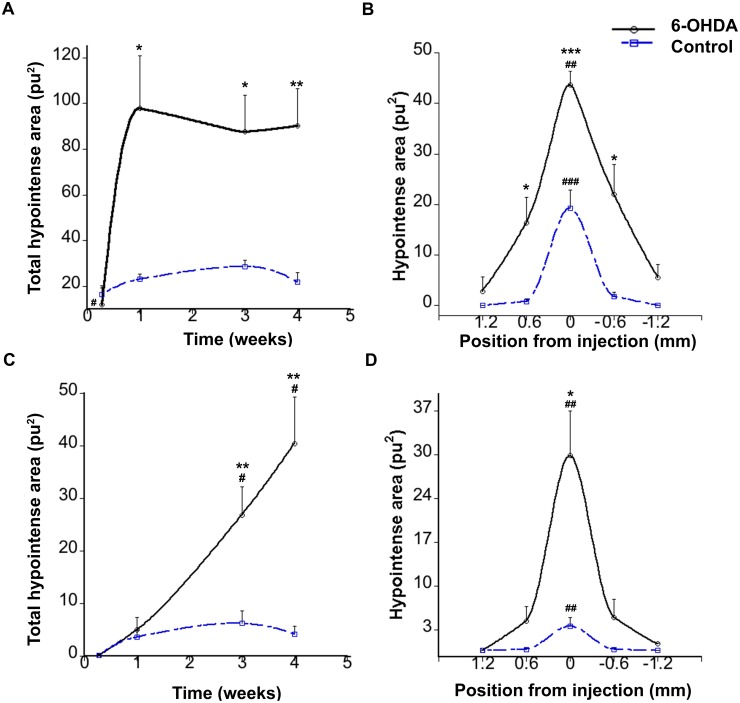
Hypointense evaluations from T2*- and T2 WI after striatal injections. A) Time course evolution of total T2*-hypointensities showed no significant differences between 1, 3 and 4 weeks, but showed differences between the two groups. B) T2*-hypointense distribution at 4 weeks expressed as distance anterior (positive) and posterior (negative) to the injection site. The results show a significant difference between 6-OHDA and control animals at different distances. C) Time course evolution of total T2-hypointensities D) T2-hypointense distribution at 4 weeks at different distances to the injection site. Continuous line represents 6-OHDA injected animals and broken line represents control animals. *p<0.05, **p<0.01, ***p<0.001 compared between control and 6-OHDA animals. ^#^p<0.05, ^##^p<0.01, ^###^p<0.001 compared between different distances or time points within each group.

To study hypointensities caused by the injection of 6-OHDA, T2*-WI and T2-WI were also performed in parallel to study the presence of hypointense areas (Fig. 4). T2*-WI were performed at different time points after 6-OHDA or vehicle injections. In 6-OHDA injected animals, T2*-WI revealed almost no hypointensities at 2 days after the lesion, instead a strong hyperintense signal was present, caused by the edema (Figs. 2 and 4A). One week postlesion, this T2*-hyperintensity was almost vanished and a hypointense signal was now visible at the place of the injection. This hypointense signal remained at least for 4 weeks (Figs. 2 and 4A). No significant differences in T2*-hypointensity values were found between the 1, 3, and 4 -week time points (F (2,10) = 1.146 p = 0.356, one-way repeated measurements ANOVA). In control animals, albeit to a lesser extent than after 6-OHDA treatment, a hypointense signal was observed as early as at 2 days (Figs. 2 and 4A), which remained constant during the time points of the study (F(2,8) = 2.392 p = 0.153, one-way repeated measurements ANOVA). T2-hypointensities at different time points were also calculated (Fig. 4C). One-way repeated measurements ANOVA showed differences in T2-hypointensities between 1, 3, 4 weeks in 6-OHDA animals (F (2,10) = 10.759 p<0.01) but not in vehicle animals (F (2,8) = 1.459 p = 0.288).

T2* and T2 hypointense areas were calculated at different distances from the injection at 4 weeks (Fig. 4 B and D respectively). The results revealed that in the 6-OHDA injected animals, the hypointense signal peaked at the injection site and spread approximately 1 mm anterior-posteriorly. On the other hand, in control animals, the hypointensity was located only to the level of the injection track (position 0.0 mm) without any further spreading. Two-way ANOVA demonstrated significant difference in the T2*-hypointense areas between 6-OHDA and control animals (F(1,45) = 41.360, *p*<0.001) with interactions between the factors (F(1,45) = 3.818, *p*<0.05). Further analyses confirmed that the hypointense areas close to the injection site had the greatest differences between the two groups (t(5.131) = 3.032 *p*<0.05, t(9) = 5.532 *p*<0.001, t(5.179) = 3.349 *p<*0.05, for coordinates 0.6, 0.0, −0.6 mm, respectively).

### Correlation between hypointensities and hyperintensities

**Figure 5:**
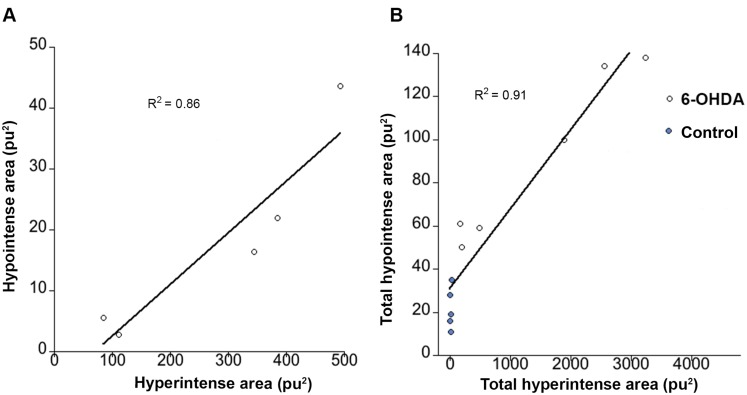
Correlations between edema and iron accumulation. A) Regression line showing the correlation between hyperintensities at day 2 and corresponding hypointensities at 4 weeks for different positions in the striatum (data points = 1.2 0.6, 0, −0.6, −1.2 mm from injection) of 6-OHDA injected animals. B) Regression line showing the correlation between total hyperintense areas at day 2 and corresponding hypointense areas at 4 weeks, calculated from control, and 6-OHDA groups (represented by blue and white dots respectively).

To demonstrate the correlation between edema and iron deposition, correlation and linear regression analyses were performed (Fig. 5). A first correlation analysis was performed to study the relation between edema and iron accumulation at different positions from the injection area. In 6-OHDA-lesioned animals, for each distance (1.2, 0.6, 0.0, −0.6, −1.2 mm from the injection site), mean hyperintense values at two days were plotted against the corresponding hypointense values at 4 weeks (Fig. 5A). Correlation for the data revealed that hypointensities and hyperintensities in the 6-OHDA were significantly related, (r = 0.92, p<0.05, two tailed). Additionally, total hyperintensities at two days were calculated for each animal in the study (control and 6-OHDA injected) and plotted against total hypointense values from the same animals at 4 weeks (Fig. 5B). The data revealed that total hypointensities and hyperintensities were correlated (r = 0.95, p<0.001 two tailed). Animals with high hyperintense values coincided with high hypointense values and vice versa.

### Hypointensities in the substantia nigra

Hypointense evaluations in the ipsilateral substantia nigra were also quantified 4 weeks after the 6-OHDA striatal injections to study the possibility of iron accumulation. The results revealed that there were no significant differences in hypointensities between control- and 6-OHDA injected animals (t (8) = 1.248 p = 0.247).

### Perls Prussian blue staining and ED1 immunohistochemistry

**Figure 6:**
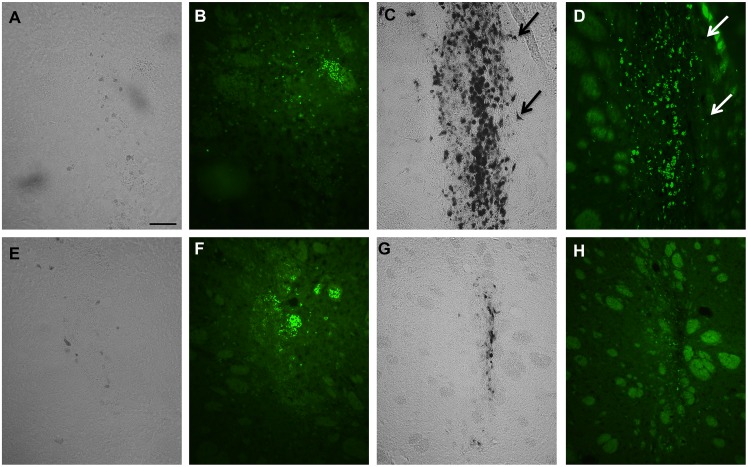
Histological analysis of hypointense areas. Prussian blue staining combined with ED1-inmunoreativity at 2 days postlesion (A, B, E, F) shows that in 6-OHDA injected animals (A, B) and control animals (E, F) iron (A and E) and microglia (B and F) are almost absent. At 4 weeks in 6-OHDA animals (C, D) the iron deposits are located to the same areas as active microglia (D). In control animals accumulation of iron (G) and activation of microglia (H) was less pronounced. Arrows show ED1-negative cells that were iron positive. Scale bar 50 µm.

**Figure 7:**
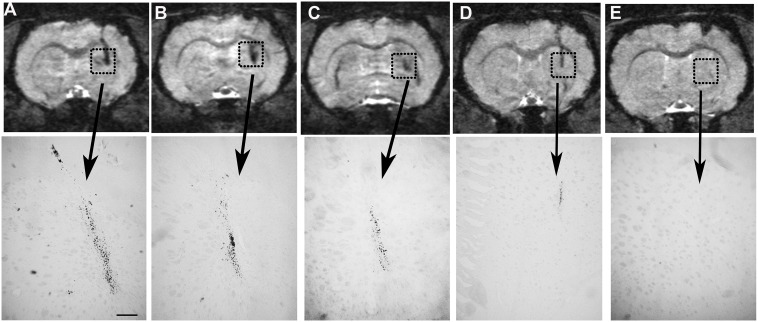
Correlations between T2*-hypointensities and iron accumulation. High resolution T2*-weighted images at four weeks after 6-OHDA injection showed a strong hypointense area at different positions in the striatum A) 0, B) −0.53 and C) −1.06 mm from the injection site which coincided with the presence of iron in Prussian blue histology. The hypointense area and the accumulation of iron was much smaller and more localized in control animals at 0 or −0.53 mm from the vehicle injection (D and E respectively). Scale bar 200 µm.

Histological Prussian blue staining of the perfused brains (control and 6-OHDA) followed by ED1 staining was performed at 2 days (Fig. 6 A, B, E, F) or 4 weeks (Fig. 6 C, D, G, H) postinjection. The results revealed that at 2 days postlesion no iron was seen in 6-OHDA animals (Fig. 6 A) or in control animals (Fig. 6E) and only a small microglia reaction was detected (Fig. 6 B, F). On the other hand, at 4 weeks postlesion, a strong accumulation of iron was seen in the 6-OHDA injected animals (Fig. 6 C) compared with control animals (Fig. 6 G). At this time point ED1 imunohistochemistry showed a strong accumulation of reactive microglia in the center of the lesioned area, which was less pronounced in control animals (Figs. 6 D and H respectively). Most of ED1-positive cells overlapped with Prussian blue-positive deposits. However, other cells than ED1-positive microglia were positive for iron (Fig. 6, arrows). The presence of iron at four weeks was correlated with the T2* hypointensities observed at different distances from the injection of 6-OHDA (Fig. 7A, B, C). In control animals the presence of iron was almost absent (Fig. 7 D, E) which accordingly also coincided with the absence of hypointensities in these animals.

### (IgG) immunohistochemistry

**Figure 8:**
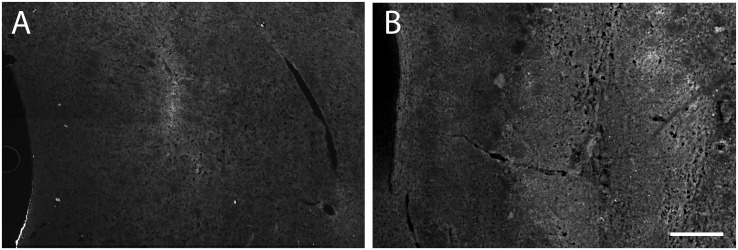
Immunoglobulin G (IgG) histochemistry. A) control- and B) 6-OHDA injected striatum at 2 days postlesion incubated with anti-rat IgG. Control animals showed almost not reactivity to IgG comparing with lesioned animals indicating that 6-OHDA provokes a disturbed BBB. Scale bar 100 µm.

The integrity of the BBB was evaluated by studying infiltration of rat IgG in the striatum of control- and 6-OHDA-injected animals (Fig. 8 A and B, respectively). Two days after performing the dopamine lesions an enhanced IgG reactivity was found in the striatum of the lesioned- compared to vehicle-injected animals. In the controls, IgG infiltration was almost absent and only a weak reaction could be observed around the vehicle injection.

### Tyrosine hydroxylase immunohistochemistry

In control animals, TH-positive nerve fibers were intact and no denervation was observed in the striatum. All animals injected with 6-OHDA showed a similar degree of nerve fiber denervation in the striatum (Figure S1).

### Giemsa staining

Giemsa staining was performed to visualize hemorrhages that might have occurred from the injections. However, no red blood cells were found in the injection tracks at 2 days postlesion (Figure S2).

## Discussion

MRI was used to follow possible iron accumulation in the intrastriatal 6-OHDA hemiparkinsonian rat. The results demonstrated that 2 days after the striatal 6-OHDA injection, a strong hyperintense signal was detected in T2-WI, which later was clearly associated with a hypointense region in T2*-WI. This hypointense area reached over several images in an anterior-posterior direction in 6-OHDA-lesioned animals while in control animals, the hypointensity was found mainly in one image, i.e. over the injection site, indicating that a larger volume was affected after the 6-OHDA lesion. The hypointensity was detected close to the injection track, in the center of the denervated area, and it was more pronounced and spread in 6-OHDA animals than in control animals. Prussian blue staining revealed accumulation of iron with similar volumetric spreading as found for the hypointensities at 4 weeks postlesion. In addition, ED-1-positive microglia was present in areas with accumulation of iron.

The presence of T2-hyperintensities after striatal injection of 6-OHDA has already been observed and described as an edematous effect caused by 6-OHDA [35]. However, the origin and nature of this edema is still under debate [35], [42], [43]. To investigate the possibility that a vasogenic edema was caused by a disrupted BBB, an immunohistochemical method was used to detect extravasated IgG [41]. The results showed that the striatum surrounding the injection trace after 6-OHDA injection was positive to antibodies against rat IgG at 2 days suggesting the existence of a vasogenic edema and an altered (BBB). In fact, the existence of a disturbed BBB in Parkinson’s disease has gained more acceptance during the last years [44] and has been proposed as one of the possible causes of neurodegeneration in Parkinson’s disease [45]. Alterations in the BBB in the striatum after 6-OHDA injections have already been described, however these studies are focused at 10 and 34 days after the dopamine lesion [46]. Thus, our results demonstrate the presence of a disrupted BBB already during the first days after the neurotoxic lesion.

Another source of the edema might origin from the neurotoxin itself. A cytotoxic edema might originate not only from the substantial loss of dopamine fibers in the striatum but also from the void of astrocytes that is evident after the 6-OHDA injection [42], [47]. Interestingly, the time profile of the edematous process seen in the present study coincides with the time determined for astrocytic death after 6-OHDA injections. The loss of astrocytes after 6-OHDA reaches a maximum at day two, and a moderate increase in astrocytes start to be detected at day eight [47]. Degeneration of astrocytes might in fact affect the permeability of the BBB to iron since, astrocytes are reinforcing the tight junctions of the blood capillaries, acting as gatekeepers for the transport of iron into the brain. Therefore, the edema might origin from a leakage in the BBB caused by the neurotoxin.

The small T2*-hypointense area found in both control and 6-OHDA-injected animals at 2 days using MRI was strictly located to the site of the injection. No iron accumulation could be found in sections stained with Prussian blue at this time point. This small hypointense area might represent hemoglobin-derived iron caused by the injection. However, neither iron accumulation nor hemorrhages were found in the histological evaluations. The small hypointensities seen at this early time point might have other explanations than accumulation of iron. Other factors than iron that may affect T2* relaxation time may include increased tissue water, altered blood flow, or myelination [28]. On the other hand the influence of other metals on T2/T2* has probably no major influence since most of metallic ions found in the brain are nonmagnetic. It has been reported that a small increase of magnetic metals such as iron, manganese, copper, or zinc occurs after injecting 6-OHDA in the vicinity of the substantia nigra [48]. Nevertheless, these amounts were considerably lower than iron, and besides, some of them, such as for example manganese have rather an effect on T1 than T2 [10]. Thus, their effect on T2 is probably negligible. Therefore the amplified hypointensity found in the striatum of 6-OHDA-injected animals at later time points was more certainly derived from iron accumulation, since iron greatly affects T2* relaxivity, and moreover the hypointensities observed in T2*-WI coincided with the presence of iron as revealed by Prussian blue staining. Although some iron could have been chelated by the presence of phosphate in the perfusion buffer, no discrepancies were found between MRI hypointensities and patterns of distribution of histological iron. In the T2-WI of the dopamine-lesioned striatum, two effects influence the image contrast. The edema will increase T2 due to the change in water content, causing a hyperintense signal, while iron accumulation will act to decrease T2, resulting in a hypointense signal. The observed contrast will be due to both of these effects and therefore the time course from the T2-WI will be difficult to interpret. The same two effects will also influence on T2* but in this case the effect of iron accumulation will be larger and we therefore believe that the time course in Fig. 4A more closely resembles the actual change in iron concentration. The differences between T2 and T2* at one week observed in the 6-OHDA injected animals are probably due to the fact that in T2-WI the hyperintensities from the edema could be detected until 1 week in most animals therefore masking the small hypointensities from the presence of iron. Thus, we believe that T2* are more reliable to measure hypointensities since there is a minor effect from the edema.

The mechanisms behind this accumulation of iron are yet not fully understood, but it might be correlated with misregulation of iron transporters [21] or the capacity of 6-OHDA to release iron from ferritin [19]. Recent studies have found the existence of iron in dopamine neuronal vesicles that are transported through the axons [49]. Thus, one source of abnormal iron accumulation in the 6-OHDA injected striatum might be the ferritin-bound iron or vesicular iron released from degenerated dopamine fibers. In addition, if the BBB is disturbed, which was the case in our study, the tight regulation of brain iron transport would be altered and as a consequence a leakage of serum iron might occur [17], [50]. Our correlation analyses between hypointense and hyperintense areas in the striatum showed an evident relation between edema formation and iron deposition. The fact that an abnormal source of iron was accumulated precisely in the area of the edema further supports the hypothesis of a disturbed BBB and a leakage of iron.

It has previously been shown that 6-OHDA can promote iron deposition in the substantia nigra when injected in the medial forebrain bundle [19], [22], [25]. In fact, treatment with iron chelators can induce neuroprotection against 6-OHDA, demonstrating a participation of iron in the neurotoxic cascade induced by 6-OHDA [51]. Therefore, quantifications of hypointensities in the substantia nigra were performed. However, due to the interference of the bone cavities it was not possible to measure using the same T2*-maps as for the striatal measurements. Thus, higher resolution images with thicker slices were used for these measurements. No differences were found in hypointensities between 6-OHDA and control animals. These results were somehow expected since the 6-OHDA striatal lesion will only cause a partial depletion of the dopamine neurons in the substantia nigra. Thus, about 60% of the dopamine neurons in the substantia nigra are reported to remain after the striatal 6-OHDA injection [40]. Therefore a more substantial depletion might be required to detect iron accumulation in the substantia nigra [19], [22], [25]. Interestingly, it has been reported that this increase of iron in the substantia nigra does not occur until 7–10 days postlesion [48]. This might in part explain our results since a delayed increase in iron occurred when 6-OHDA was injected in the striatum.

Histological analyses using Prussian blue and ED1-immunohistochemistry, revealed an area of reactive microglia that was restricted to the region of iron accumulation. Other cells that were ED1-negative also seemed to accumulate iron. Thus, iron accumulation was not exclusively restricted to ED1- positive cells. It is known that iron laden-microglia has been found in the brains of Parkinson’s disease patients [52], 6-OHDA-induced animals [25], or other neurodegenerative diseases [53], suggesting a pathological role for iron-laden microglia. Therefore, the presence of activated microglia in the lesioned striatum probably represents an area where phagocytosis occurs induced by tissue damage and an excess of iron. The fact that iron accumulation and hypointensities were restricted to the same area as ED1 suggest that iron-laden microglia might account for a significant part of the MRI hypointensities.

## Conclusions

The present study demonstrates by using *in vivo* MRI that iron accumulates in the striatum of a rat model of early Parkinson’s disease where slow, retrograde neurodegeneration occurs. Injection of 6-OHDA in the rat striatum results in an edematous process accompanied with an abnormal accumulation of iron. In addition, this excess of iron is located in the same area as active microglia, suggesting a pathological role for this accumulation of iron. The association of iron overload with an edematous process suggests the presence of a vasogenic edema and a disrupted BBB as possible sources for this excess of iron.

## Supporting Information

Figure S1 **Tyrosine hydroxylase immunoreactivity.** A) Control- and B) 6-OHDA injected striatum at 4 weeks after injections showing dopaminergic integrity or degeneration, respectively, around the injection track (black arrows). The size of the denervated zone appears similar in all lesioned animals. A and B represent composite images. Scale bar 200 µm.
(TIF)

Figure S2 **Giemsa staining.** A) control- and B) 6-OHDA injected striatum at 2 days postlesion incubated with Giemsa stain showed absence of red blood cells in both control- and 6-OHDA lesioned animals. Scale bar 200 µm.
(TIF)
