# Supplementary figures and images for: Magnetic Resonance Imaging (MRI) to Study Striatal Iron Accumulation in a Rat Model of Parkinson’s Disease
Source: PLoS One. 2014 Nov 14;9(11):e112941. doi: 10.1371/journal.pone.0112941 (PMC4232582; doi:10.1371/journal.pone.0112941)

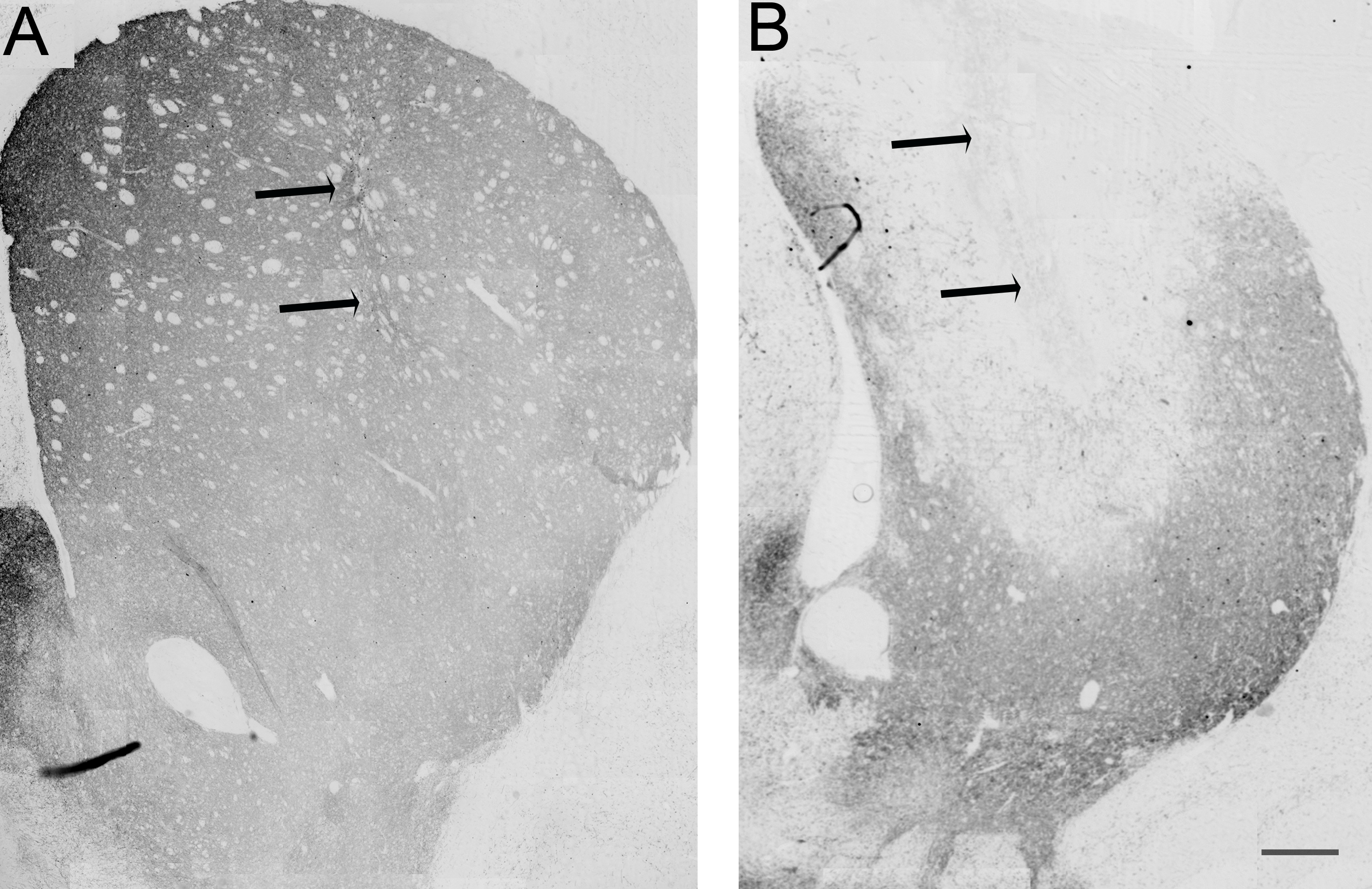

Supplement: Figure S1 — Tyrosine hydroxylase immunoreactivity. A) Control- and B) 6-OHDA injected striatum at 4 weeks after injections showing dopaminergic integrity or degeneration, respectively, around the injection track (black arrows). The size of the denervated zone appears similar in all lesioned animals. A and B represent composite images. Scale bar 200 µm. (TIF) [file pone.0112941.s001.tif]

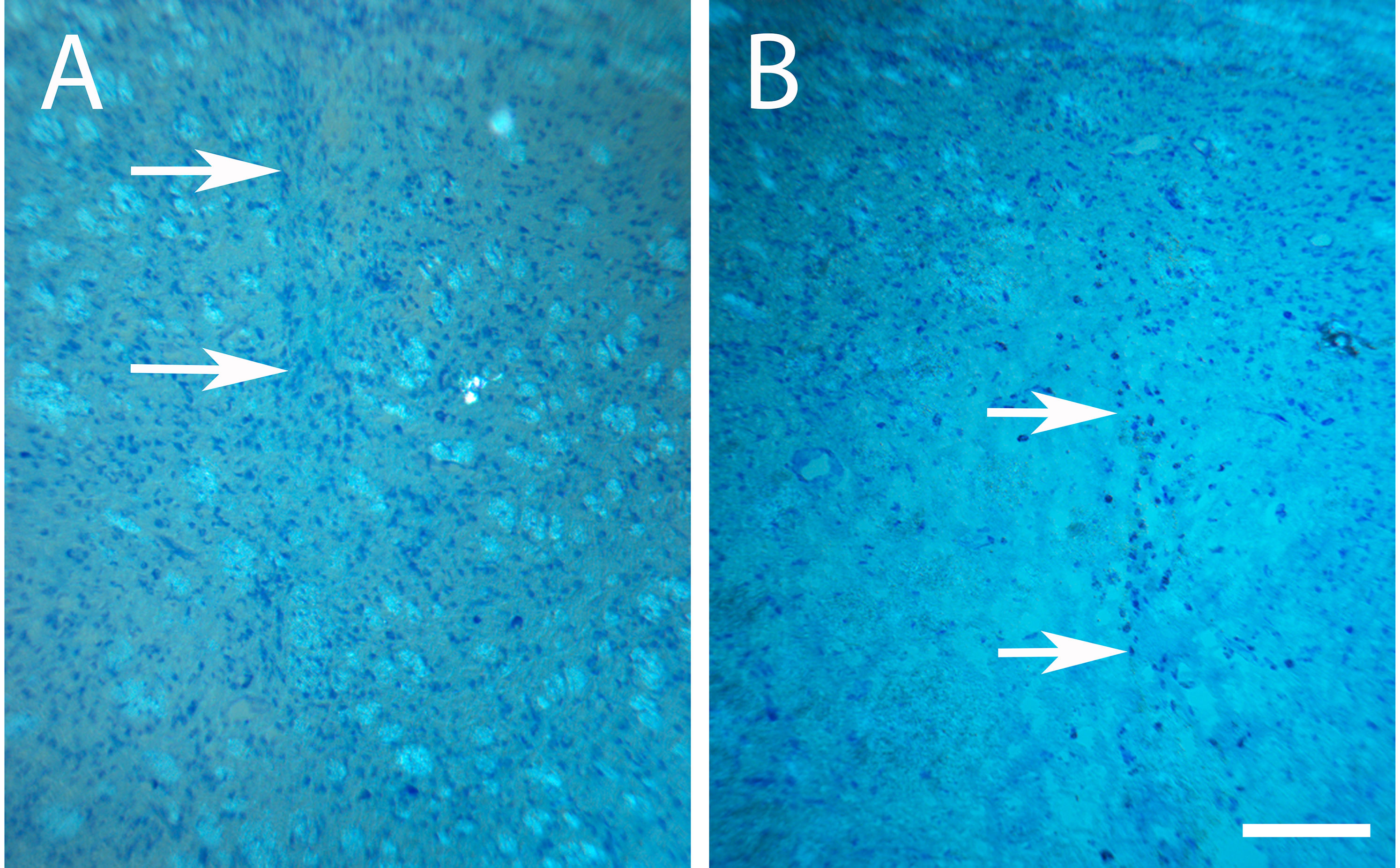

Supplement: Figure S2 — Giemsa staining. A) control- and B) 6-OHDA injected striatum at 2 days postlesion incubated with Giemsa stain showed absence of red blood cells in both control- and 6-OHDA lesioned animals. Scale bar 200 µm. (TIF) [file pone.0112941.s002.tif]
